# Gram-scale synthesis of FICZ, a photoreactive endogenous ligand of the aryl hydrocarbon receptor

**DOI:** 10.1038/s41598-019-46374-7

**Published:** 2019-07-10

**Authors:** Cunyu Zhang, Katrina L. Creech, William J. Zuercher, Timothy M. Willson

**Affiliations:** 1 Platform Technology Sciences, GlaxoSmithKline, Collegeville, PA USA; 2 Structural Genomics Consortium, UNC Eshelman School of Pharmacy, University of North Carolina at Chapel Hill, Chapel Hill, NC USA

**Keywords:** Small molecules, Toxicology

## Abstract

Development of an efficient and scalable synthesis of 6-formylindolo[3,2-b]carbazole (FICZ), a naturally-occurring aryl hydrocarbon receptor (AhR) ligand, allowed its biological and physical properties to be studied. FICZ was shown to be the most potent among a series of 6-substituted indolo[3,2-b]carbazoles for activation of AhR in cells. Photostability studies of FICZ revealed a non-enzymatic mechanism for its conversion to a biologically active quinone. These results further support the hypothesis that FICZ is a light-dependent hormone that links sun exposure to regulation of biological pathways in peripheral tissues.

## Introduction

**Figure 1:**
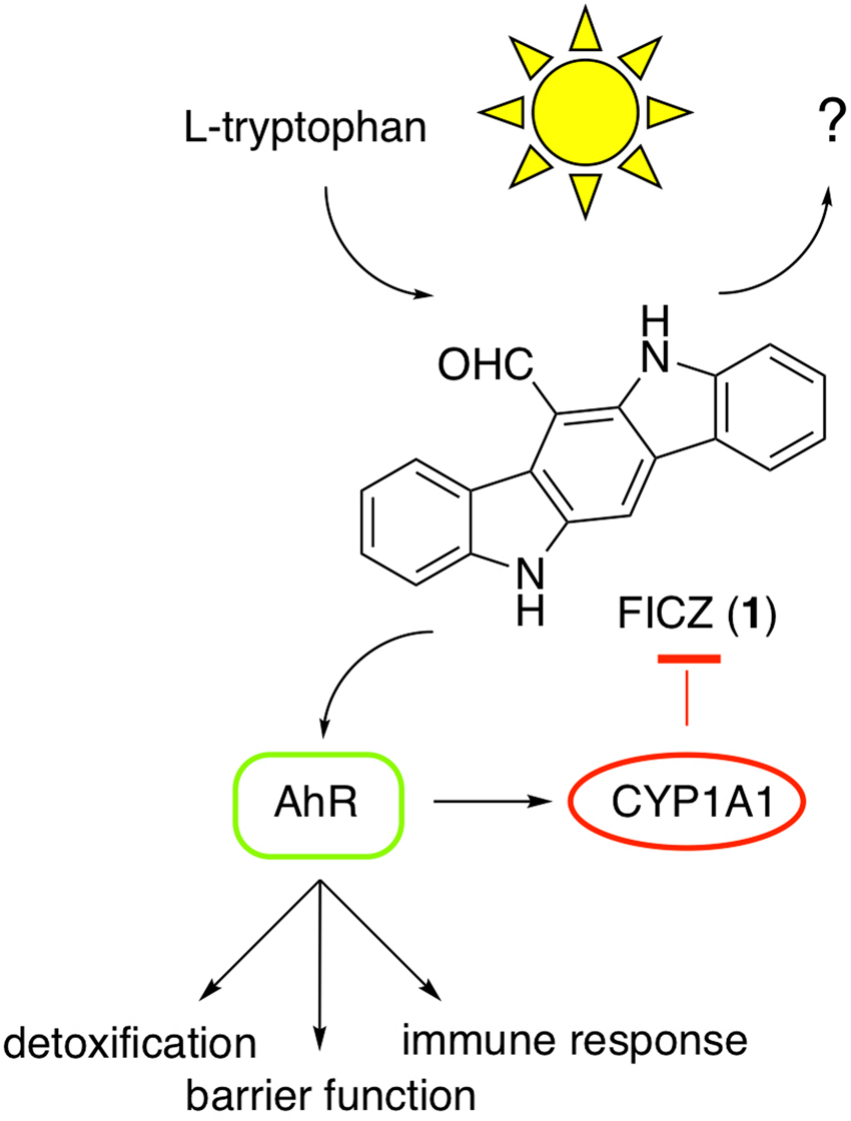
Light dependent synthesis of FICZ. 6-Formylindolo[3,2-b]carbazole (FICZ, **1**) is formed by the action of light on tryptophan. FICZ activation of AhR regulates important biological pathways and increases expression of CYP1A1 as a feedback mechanism to induce its own metabolism.

6-Formylindolo[3,2-b]carbazole (FICZ, **1**) is an important photoproduct of tryptophan (Fig. 1) that was isolated by the Rannug laboratory as a light-induced ligand for the aryl hydrocarbon receptor (AhR)^1^. FICZ (**1**) is one of the most potent naturally-occurring AhR ligands. Strong evidence for its formation in humans by the action of light on the skin and its metabolism by the AhR-inducible CYP1A have been presented^2–4^. As such, FICZ (**1**) fulfills many of the requirements for a hormone that links sunlight to regulation of peripheral biological rhythms^5^. Conversion of tryptophan to FICZ (**1**) in cells requires the action of visible or UV-light and is accelerated by the presence of hydrogen peroxide^6^. Notably, FICZ (**1**) in combination with UV-light also leads to the induction of oxidative stress and dermal toxicity^7^.

Despite increased interest in the study of FICZ (**1**) as light-dependent hormone with potential immune-modulating activity^8,9^, it is available in only limited quantities and at high cost from commercial vendors. The chemical synthesis of FICZ (**1**) was first described by Bergman using a low yielding route that required purification of the final product by sublimation under reduced pressure^10^, although subsequent improvements were made to facilitate the synthesis of several related carbazoles^11,12^. More recent syntheses were also completed on scales yielding only a few milligrams of product^13,14^. A key challenge in the synthesis is the difficult purification of the carbazole intermediates due to their poor solubility. To address the issue that no efficient large-scale synthesis FICZ (**1**) has been reported to date^15^, we developed an optimized procedure to produce it in gram quantities. We also determined the biological activity of several intermediate 6-substituted indolo[3,2-b]carbazoles that illuminated the critical role of the 6-formyl group in activation of AhR. Finally, the availability of gram quantities of FICZ (**1**) led to the discovery of a previously unknown photo-decomposition pathway that may contribute to its biological role as a light-dependent hormone (Fig. 1).

## Results

### Practical synthesis of FICZ (1)

**Figure 2:**
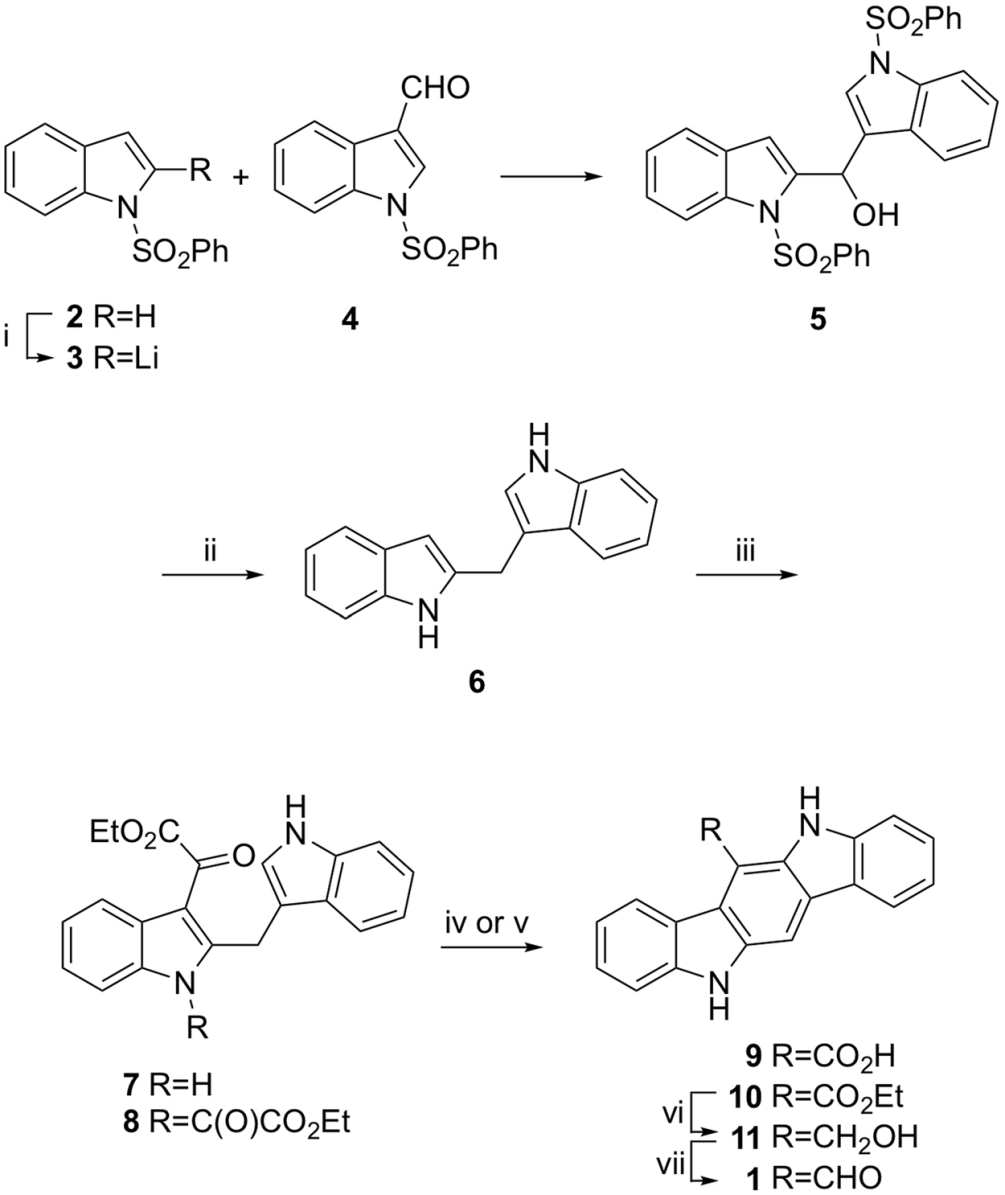
Practical chemical synthesis of FICZ (1). (i) *n*-BuLi, THF, −70 °C. 77%. (ii) LiAlH_4_, THF, reflux. (iii) EtO_2_CCOCl, pyridine, THF. 52% for two steps. (iv) 4N NaOH, dioxane, 90 °C; 1N HCl, 100 °C. ~50%. (v) MeSO_3_H, dioxane, reflux. 99%. (vi) BH_3_•Me_2_S, THF, reflux. 89%. (vii) DDQ, dioxane. 90%.

The readily available 1-(phenylsulfonyl)-1H-indole (**2**, Fig. 2) was selected as one of the starting materials, since the phenylsufonyl group protecting group would direct ortho-lithiation of the indole by *n*-BuLi^16^. Reaction of the 2-lithiate salt **3** with commercially available 1-(phenylsulfonyl)-1H-indole-3-carbaldehyde (**4**) afforded the desired bis-indole intermediate **5** in a good yield following chromatography.

Removal of the phenylsulfonyl groups and benzylic hydroxyl was achieved by heating with excess lithium aluminum hydride followed by a standard Fieser work up protocol to afford crude **6** at 80% purity. The limited stability of **6**^11^ required use of the crude material without further purification. Reaction of **6** with excess ethyl oxalyl chloride did not yield the desired 3-acylated indole **7** but instead produced the double-acylated product **8** due to the NH group reacting as a vinylogous amide. Saponification of the crude **8** in 4N NaOH followed by heating in aqueous HCl gave a low yield of the known carboxylic acid CICZ (**9**). Fortunately, reaction of crude **6** with 0.95 equivalents of ethyl oxalyl chloride yielded pure mono-carboxy **7** in good yield following crystallization from acetonitrile. Ring closure and carbazole formation was achieved by heating **7** in dioxane with methanesulfonic acid followed by work up and crystallization to deliver pure carbazole ester **10**. Borane-methyl sulfide complex was found to be the optimal reagent for reduction of the ester **10** due to the homogenous reaction mixture and simple work up to produce alcohol **11**. Oxidation of **11** by DDQ yielded aldehyde **1**, which was isolated by filtration. Following extensive washing with water, analytically pure FICZ (**1**) was produced as a yellow solid in high yield. The synthetic procedure (Fig. 2) was successfully run on a multi-gram scale. Purification of the ring-closed carbazoles by crystallization was critical, since their poor solubility made column chromatography on large scale impractical.

### AhR activation by 6-substituted indolo[3,2-b]carbazoles

Although several indolo[3,2-b]carbazoles have been reported as AhR ligands^2,17^, there has been no systematic study of their relative activity as activators of the receptor. AhR ligands cause translocation of the receptor to the cell nucleus where the AhR-ligand complex binds to the promoter region of the *cyp1a1* gene to increase its transcription (Fig. 1). We employed a commercially available assay for AhR ligands in which a cell line had been engineered to express a reporter gene composed of the *cyp1a1* promoter driving expression of a β-lactamase reporter. AhR ligands were detected by the increased expression of β-lactamase, which was quantified using a fluorescent substrate (see *Materials and methods*). In this assay, the standard AhR ligand TCDD demonstrated an EC_50_ = 0.3 nM (data not shown). Using the *cyp1a1* gene reporter assay, we determined the AhR agonist activity of FICZ (**1**) and several of its 6-substituted analogs (Table 1). FICZ (**1**) was the most potent analog with an average EC_50_ of 10 nM, consistent with its reported potency for induction of IL-22 and inhibition of IL-17 in immune cells^18,19^. The unsubstituted carbazole ICZ (**12**), a known AhR ligand that is generated in the gut from dietary sources^20^, was only slightly less potent. The other three 6-substituted analogs (**9**–**11**) were much less potent than FICZ (**1**). Notably, CICZ (**9**) a naturally-occurring oxidation product of FICZ (**1**)^2^ was 40-times less potent as an AhR agonist.

**Table 1:** AhR activity of 6-substituted indolo[3,2-b]carbazoles and quinone (**13**).


Compound	R	AhR EC_50_ (nM)
FICZ (**1**)	CHO	10 ± 10
CICZ (**9**)	CO_2_H	400 ± 100
**10**	CO_2_Et	160 ± 40
**11**	CH_2_OH	160 ± 90
ICZ (**12**)	H	30 ± 10
**13**		70 ± 15

Compounds were compared in a *cyp1a1* reporter gene assay. The EC_50_ for activation of AhR was determined, n = 4 ± S.D.

### Photostability of FICZ

**Figure 3:**
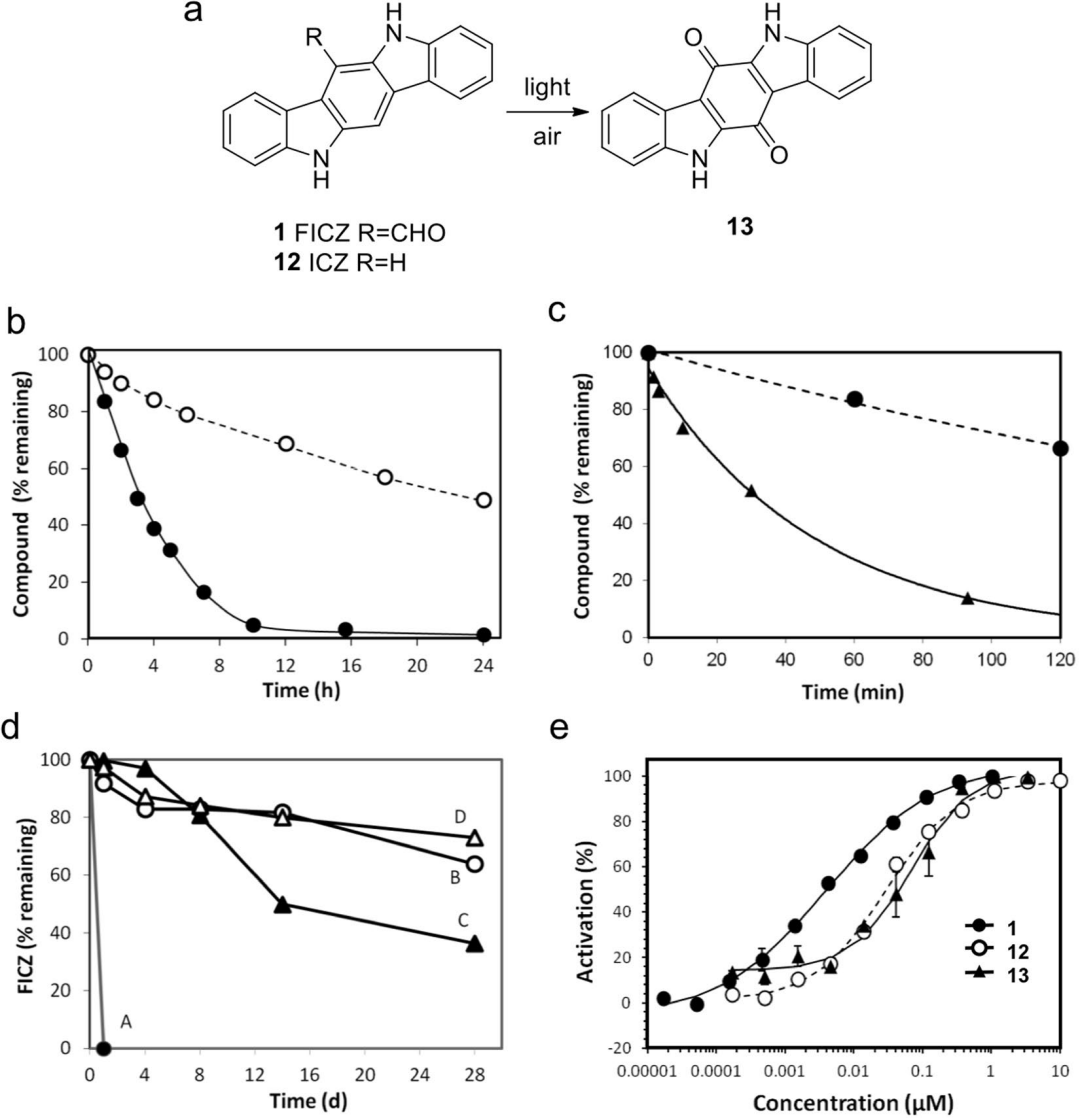
FICZ photostability (**a**) FICZ (**1**) and ICZ (**12**) decompose to quinone (**13**) (**b**) Time course of FICZ (**1**) ● and ICZ (**12**) О stability in air and bright light (**c**) Time course of FICZ (**1**) stability in air and bright light ● or direct sunlight ▲ (**d**) FICZ (**1**) stability in (A) air and light ●, (B) nitrogen and light О, (C) air and dark ▲, and (D) nitrogen and dark △ (**e**) Representative data from a duplicate experiment measuring activation of the human AhR in a *cyp1a1* reporter gene assay by a freshly prepared sample of FICZ (**1**), ICZ (**12**), or quinone (**13**).

Although FICZ (**1**) was stable as a pure solid, we observed decomposition of DMSO solutions upon standing under laboratory lighting. To more accurately measure the photostability of **1** by ^1^H NMR, a deuterated DMSO solution was placed in a clear Flint bottle inside a closed chamber and subjected to a light intensity of 11000 lux (approximately equivalent to daylight without direct sun exposure). Maleic acid was employed as an internal NMR reference. FICZ (**1**) decomposed rapidly in the presence of air and light with a half-life of around 3 h (Fig. S1). Only a single carbazole product could be observed by ^1^H NMR over the time course of the experiment. Repetition of the photo-reaction on an 80 mg scale over 10 d also yielded a single cabazole product that was identified as the quinone indolo[3,2-b]carbazole-6,12-dione (**13**) by MS, ^1^H and ^13^C NMR (Fig. 3a and S1)^21^. When the unsubstituted carbazole ICZ (**12**) was included as a comparator, decomposition was at least 10-times slower under identical conditions (Fig. 3b) and the same quinone (**13**) was formed as supported by ^1^H NMR and LCMS analysis. When the experiment was repeated in direct sunlight at a light intensity of 63000 lux, decomposition of FICZ (**1**) was even more rapid with a half-life of ~30 min (Fig. 3c). To further characterize the stability of FICZ (**1**) and explore the mechanism of photo-decomposition, DMSO solutions were subjected to 11000 lux over 28 d under different storage conditions (Fig. 3d). Significant decomposition was observed under all conditions, however the rate varied depending on the presence of bright light or air. Replicating the prior conditions of bright light and air, FICZ (**1**) was completely decomposed within 1 day (Condition A). When the bottle was flushed with nitrogen to minimize the presence of air, decomposition of FICZ (**1**) was much slower (Condition B). In contrast, protection of the bottle from light exposure with aluminum foil further slowed, but did not prevent the decomposition (Condition C). Finally, removal of air and protection from light (Condition D) yielded the slowest rate of decomposition. Since the photo-reaction was highly dependent on the presence of air and light, we propose that the formation of quinone (**13**) from FICZ (**1**) proceeds by a radical chain reaction through an intermediate carbazole 6-carboperoxy radical (B, Fig. S2). Although we were unable to observe any reactive intermediates by ^1^H NMR (Fig. S1), a peak potentially corresponding to CICZ (**9**) (from intermediate C in Fig. S2) was occasionally observable by LCMS but could not be isolated (data not shown). In summary, FICZ (**1**) showed a wide range of stability as a solution in DMSO. Decomposition required the presence of air and was greatly accelerated by bright light. Importantly, we observed that air or light alone was sufficient to promote significant decomposition of FICZ (**1**) as a DMSO solution upon prolonged storage.

### Bioactivity of FICZ and its photoproduct

Using the *cyp1a1* reporter assay to determine the potency for AhR activation, the photo-decomposition product quinone (**13**) showed an EC_50_ value of 70 nM (Table 1). Thus, photo-decomposition of FICZ (**1**) to the quinone (**13**) results in a ligand with at least 7-fold decreased potency for the activation of AhR (Table 1 and Fig. 3e). It remains to be determined whether this difference would to be biologically significant.

## Discussion

FICZ (**1**) demonstrates many of the properties of a light-dependent hormone that could entrain peripheral tissues through exposure to sunlight. FICZ (**1**) is formed by UV or light irradiation of tryptophan^22^ and, following activation of AhR, can increase expression of CYP1A1 to induce its own metabolism^4,23^. Our photostability experiments now reveal an alternative pathway to limit exposure of peripheral tissues to high levels of FICZ (**1**) through a non-enzymatic light and air-induced oxidation of the indolocarbazole.

Notably, photodecomposition of FICZ (**1**) was much more rapid than the unsubstituted analog ICZ (**12**), an alternate AhR ligand that is formed in the gut from dietary precursors but is not produced by light^15^. Although both naturally-occurring indolocarbazoles decomposed to the identical quinone (**13**), the presence of the aldehyde in FICZ (**1**) clearly accelerated photo-induced oxidation of the carbazole core. Aromatic aldehydes undergo photo-oxidation much more rapidly than their corresponding unsubstituted analogs likely due to the formation of intermediate benzoylperoxy radicals^24^. Photo-oxidation of FICZ (**1**) may proceed by an analogous radical chain process, given its dependency on light and air (Fig. S2). The generation of radical intermediates and propagation of the radical chain would be facilitated by the aldehyde in FICZ (**1**), whereas radical initiation from ICZ (**12**) would be much slower. However, this mechanism is only speculative given the absence of stable intermediates that were isolated in our studies.

Our stability experiments were conducted using light intensities comparable to normal daylight as well as in direct bright sunlight. We observed that photodecomposition was faster in the brighter light corresponding to direct sun exposure. Although regular laboratory lighting is generally 20–100 times less intense than daylight, our results dictate that solutions of FICZ (**1**) should be prepared immediately prior to biological testing and be shielded from air and light to ensure accurate results.

Although FICZ (**1**) was first identified as a potent AhR ligand over 20 years ago^1^, there have been no systematic studies of its structure-activity for activation of the receptor. The reason may be due, in part, to a lack of an efficient synthesis of FICZ analogs. With access to a series of 6-substituted analogs, generated as intermediate compounds in our improved synthesis, we employed a cell-based reporter assay to explore the role of this key substituent. *Cyp1a1* is a primary target gene of AhR in cells (Fig. 1). Using a commercial assay where the *cyp1a1* promoter drives expression of a ß-lactamase, we found that FICZ (**1**) and the unsubstituted carbazole ICZ (**12**) showed similar potency in cells. In contrast, the 6-substituted acid (**9**), ester (**10**), and alcohol (**11**) were each less active. Thus, the 6-formyl group in FICZ (**1**) appears to be the only one of these substituents that is well tolerated by the receptor. Although the quinone (**13**) was slightly less active than FICZ (**1**), it was still more potent as an AhR agonist than the other 6-substituted carbazoles.

FICZ (**1**) is a naturally-occurring AhR ligand that is formed in the skin^3,25^ and may be an important physiological regulator of the transcription factor^9^. Many dermatological conditions, such as atopic dermatitis and psoriasis, can be alleviated though judicious sun exposure^26^. In fact, therapeutic UV-B dosing is a common treatment for both conditions. FICZ (**1**) has demonstrated immune modulating effects through activation of AhR and is effective in treating animal models of psoriasis^27^. Pharmacological treatment of atopic dermatitis and psoriasis with topical AhR ligands has also been shown to be effective in human clinical trials^28,29^. Thus, the daily light-regulated level of FICZ (**1**) may have an important role in maintaining healthy skin^30^. Our results suggest the possibility that the combination of light exposure with free oxygen in cells provides an additional non-enzymatic mechanism for limiting the total levels of FICZ (**1**) in peripheral tissues.

Quinones are highly redox active molecules that redox cycle with semiquinone radical anions resulting in the formation of hydrogen peroxide and reactive oxygen species (ROS). Electron rich quinones with extended ring systems, such as **13**, are known to be efficient substrates for redox cycling (Fig. S2)^31^. It is theoretically possible that photodecomposition of FICZ (**1**) to quinone (**13**) is part of a feedback system to protect skin from photoaging due to oxidative stress. It is notable that FICZ (**1**) is a photosensitizer that increases ROS levels in cells exposed to UVA^7^. Blue light has also been shown to increase H_2_O_2_ levels in cells and activate AhR signaling^32^. Our results raise the possibility that formation of quinone (**13**) may contribute to this process. FICZ (**1**) and its photoproduct (**13**) merit further research to explore their roles in the photobiology of skin health and aging due to sunlight.

In conclusion, we have developed the first efficient gram-scale synthesis of FICZ (**1**), which will allow investigators ready access to this important tool for the study of AhR biology in pharmacological models of disease. Our results provide support for the proposed role of FICZ (**1**) as a light-dependent switch for a ligand-dependent transcription factor in peripheral tissues, such as the skin. It is remarkable that nature has non-enzymatic light-facilitated mechanisms to both generate^1,2^ and remove this signaling molecule. Whether this process functions in humans to limit the concentration of FICZ (**1**) in skin under conditions of bright sunlight or protect the skin from the detrimental effects of ROS through formation of quinone (**13**) remains to be explored.

## Materials and Methods

Starting materials, reagents, and solvents were obtained from commercial sources and used as received. ICZ was purchased from AstaTech. Intestinal human colon adenocarcinoma LS180 cells stably transfected with a β-lactamase reporter gene downstream of the *cyp1a1* promoter were obtained from Invitrogen (catalog number K1678) and were used in accordance with the protocol described at https://www.thermofisher.com/document-connect/document-connect.html?url=https%3A%2F%2Fassets.thermofisher.com%2FTFS-Assets%2FLSG%2Fmanuals%2Fgeneblazer_cyp1a1blals0180_man.pdf, but adapted to run in 384-well plates.

^1^H NMR and proton decoupled ^13^C NMR spectra were recorded on a Bruker 400-MHz spectrometer in CDCl_3_ or DMSO-*d*_6_ with TMS as the internal reference. Chemical shifts (δ) are reported in parts per million (ppm) relative to deuterated solvent as the internal standard (δH: CDCl_3_ 7.26 ppm, DMSO-*d*_6_ 2.50 ppm), coupling constants (J) are in hertz (Hz). Peak multiplicities are expressed as follows: singlet (s), doublet (d), doublet of doublets (dd), triplet (t), quartet (q), multiplet (m), and broad singlet (br s). Melting points were recorded with a Büchi 520 apparatus and are uncorrected. Purification of intermediates and final products, if mentioned, was carried out on normal phase using an ISCO CombiFlash system and prepacked SiO_2_ cartridges eluted with optimized gradients of heptane−ethyl acetate mixture. Progress of the reactions was monitored by thin-layer chromatography (TLC) analysis (Merck, 0.2 mm silica gel 60 F254 on glass plates) or by LCMS (described below). The purity of all products was established on a LCMS method:

LC conditions: UPLC analysis was conducted on a Phenomenex Kinetex 1.7 um XB-C18 column at 40 °C. 0.2 uL of sample was injected using a partial loop with needle overfill injection mode. The gradient employed was: mobile phase A – water +0.2% v/v formic acid., mobile phase B – acetonitrile +0.15% v/v formic acid. UV detection was provided by summed absorbance signal from 210 to 350 nm scanning at 40 Hz. MS detection by Waters SQD LBA555 using alternating positive/negative electrospray with scan range = 125–1200 amu, scan time = 110 msec, and interscan delay = 50 msec.

### (1-(Phenylsulfonyl)-1H-indol-2-yl)(1-(phenylsulfonyl)-1H-indol-3-yl)methanol(5)

To a stirred solution of 1-(phenylsulfonyl)-1H-indole (9.02 g, 35.0 mmol) in dry 2-methyltetrahydrofuran (200 mL) at −78 °C was added 1.6 M *n*-butyllithium in hexane (28 mL, 44.8 mmol, 1.28 eq.) slowly over 15 min. The mixture was warmed to room temperature and stirred for 0.5–1 h. The reaction mixture became a slurry. It was cooled back to −78 °C and a solution of 1-(phenylsulfonyl)1-(phenylsulfonyl)-1H-indole-3-carbaldehyde (10 g, 35 mmol, 1.0 eq.) in 110 mL of THF was added dropwise over 20 min. The reaction mixture became a homogeneous solution. After stirring for 30 min with continued cooling by a dry ice-acetone bath, LCMS revealed the aldehyde had been consumed. The reaction was quenched by slowly adding 1 N HCl aq. solution (100 mL) and then allowed to warm to room temperature and stirred overnight. The reaction mixture was diluted with EtOAc (200 mL) and brine (200 mL). The organic was separated, and the aqueous phase was further extracted with EtOAc (100 mL). The combined organic (volume ~600 mL) was washed with brine, dried over MgSO_4_ and concentrated under reduced pressure to yield a crude brown liquid (81% purity) that was purified by chromatography on a 120 g ISCO silica gel column (20–40% EtOAc/heptane) to afford the title compound as a yellow foam (15.4 g, 77% yield). ^1^H NMR (400 MHz, CDCl_3_) δ: 8.17 (1 H, d, J = 9.0 Hz), 8.03 (1 H, d, J = 8.5 Hz), 7.92–7.97 (2 H, m), 7.79–7.85 (2 H, m), 7.74 (1 H, d, J = 0.75 Hz), 7.55–7.62 (2 H, m), 7.43–7.51 (4 H, td, J = 7.87 and 15.6 Hz), 7.30–7.38 (3 H, m), 7.21–7.27 (1 H, m), 7.07 (1 H, t, J = 7.28 Hz), 6.94 (1 H, d, J = 8.03 Hz), 6.55 (1 H, d, J = 5.02 Hz), 6.23 (1 H, s), 3.71 (1 H, d, J = 5.27 Hz). LCMS m/z: [M-OH]^+^  = 525.53, rt = 1.02 min.

### Ethyl 2-(2-((1H-indol-3-yl)methyl)-1H-indol-3-yl)-2-oxoacetate (7)

To a stirred suspension of lithium aluminum hydride (9.42 g, 248 mmol, 9.05 eq.) in dry 2-methyltetrahydrofuran (250 mL) at −20 °C was added slowly a solution of (1-(phenylsulfonyl)-1H-indol-2-yl)(1-(phenylsulfonyl)-1H-indol-3-yl)methanol (15.4 g, 27.4 mmol) in 100 mL of 2-methyltetrahydrofuran. The flask was covered with aluminum-foil, heated under gentle reflux for 6.5 h and then stirred at room temperature overnight. LCMS revealed the starting material (rt = 1.02 min) was nearly consumed and the product at rt = 0.89 min was a major peak. The reaction mixture was cooled to 0 °C, quenched by adding slowly 9.4 mL of water, 9.4 mL of 15% NaOH and 28.2 mL of water sequentially, stirred for 1 h and filtered through a Celite pad. The filter cake was washed with EtOAc (~500 mL). The filtrate was washed with brine two times, quickly dried over MgSO_4_ and concentrated under reduced pressure to an amber oil that was used without further purification. A solution of the resulting crude 3-((13-((1H-indol-2-yl)methyl)-1H-indole (7.85 g, 25.5 mmol based on 80% purity by LCMS) and pyridine (3.09 mL, 38.2 mmol, 1.5 eq.) in dry THF (100 mL) was covered with aluminum foil and cooled to 0 °C. Ethyl oxalyl chloride (2.70 mL, 24.1 mmol, 0.95 eq.) was dissolved in 25 mL of THF and added dropwise over a period of 60 min. The mixture was stirred at room temperature overnight and then partitioned between CH_2_Cl_2_ (250 mL) and 5% sodium bicarbonate (100 mL). The organic layer was separated, washed with brine, dried over MgSO_4_ and concentrated under reduced pressure. The residue was recrystallized in acetonitrile (~75 mL) to afford the title compound as a pale yellow solid (3.65 g). The filtrate was concentrated to half volume to provide a second batch (0.95 g). Total = 4.60 g, 52% yield. ^1^H NMR (400 MHz, CDCl_3_) δ: 8.44 (1 H, br s), 8.28 (1 H, br s), 7.97 (1 H, d, J = 8.0 Hz), 7.50 (2 H, d, J = 8.5 Hz), 7.24–7.33 (3 H, m), 7.13–7.23 (3 H, m), 4.69 (2 H, s), 4.51 (2 H, q, J = 7.3 Hz), 1.46 (3 H, t, J = 7.2 Hz). LCMS m/z: [M + H]^+^  = 347.34, rt = 0.85 min.

### 5,11-Dihydroindolo[3,2-b]carbazole-6-carboxylic acid (9)

Ethyl 2-(3-((3-(2-ethoxy-2-oxoacetyl)-1H-indol-2-yl)methyl)-1H-indol-1-yl)-2-oxoacetate (0.5 g, 39% purity) was dissolved in 1,4-dioxane (20 mL). 4 N sodium hydroxide (5.0 mL, 20.0 mmol, 45.8 eq.) was added. The resulting mixture was heated at 90 °C for 3 h and cooled to room temperature. The organic solvent was removed in vacuo. The insoluble solids were removed by filtration. The filtrate was adjusted to pH 3–4 with 1 N HCl. A large amount of solid precipitated. LCMS revealed the cyclization had occurred, but was not complete. The suspension was heated at 90–100 °C for 1 h and cooled in an ice-water bath. The tan solid was collected by filtration, washed with water and dried to afford the crude title product. The crude material was dissolved in EtOAc and purified on a silica gel chromatography column (50% EtOAc/heptane). The solid obtained was suspended in hot MeOH, cooled, filtered, washed with MeOH and dried to afford the pure title compound as a yellow solid (100 mg, ~50% yield). ^1^H NMR (400 MHz, DMSO-d_6_) δ 13.53 (s, 1 H), 11.48 (s, 1 H), 10.99 (s, 1 H), 8.93 (d, *J* = 8.28 Hz, 1 H), 8.46 (s, 1 H), 8.26 (d, *J* = 7.53 Hz, 1 H), 7.72 (d, *J* = 8.28 Hz, 1 H), 7.53 (d, *J* = 8.03 Hz, 1 H), 7.42 (t, *J* = 7.65 Hz, 2 H), 7.05–7.25 (m, 2 H). LCMS m/z: [M-H]^+^  = 299.24, rt = 0.79 min.

### Ethyl 5,11-dihydroindolo[3,2-b]carbazole-6-carboxylate (10)

Into a solution of ethyl 2-(2-((1H-indol-3-yl)methyl)-1H-indol-3-yl)-2-oxoacetate (4.99 g, 14.4 mmol in 1,4-dioxane (150 mL) was added slowly methanesulfonic acid (7.5 mL, 115 mmol, 8.0 eq.). The resulting mixture was heated at 105 °C under gentle reflux for 30 min. LCMS revealed complete conversion to a single product. The mixture was cooled and concentrated under reduced pressure to a minimal volume. The residue was suspended in 5% NaHCO_3_ (250 mL) and stirred for 1 h. The solid was collected by filtration, washed with EtOAc and water, and dried in air flow to afford the title compound as a yellow solid (3.72 g). The organic was separated from the filtrate, washed with brine, dried over MgSO_4_ and concentrated under reduced pressure to give a second batch of the title compound (1.0 g). Total = 4.72 g, 99% yield. mp 175–177 °C (decomp.). ^1^H NMR (400 MHz, DMSO- *d*_6_) δ: 11.51 (1 H, s), 10.95 (1 H, s), 8.72 (1 H, d, J = 8.3 Hz), 8.49 (1 H, s), 8.28 (1 H, d, J = 7.8 Hz), 7.69 (1 H, d, J = 8.0 Hz), 7.54 (1 H, d, J = 8 Hz), 7.45 (2 H, m), 7.19 (2 H, m), 4.69 (2 H, q, J = 7.2 Hz), 1.53 (3 H, t, J = 7.2 Hz). ^13^C NMR (100 MHz, DMSO-*d*_6_) δ: 167.5, 142.1, 141.6, 136.2, 135.6, 126.7, 126.6, 125.4, 123.8, 122.1, 121.7, 120.8, 120.5, 119.1, 118.2, 112.0, 111.2, 107.2, 105.6, 61.5, 14.5. LCMS m/z: [M + H]^+^  = 329.52, rt = 0.99 min.

### (5,11-Dihydroindolo[3,2-b]carbazol-6-yl)methanol (11)

Into a solution of ethyl 5,11-dihydroindolo[3,2-b]carbazole-6-carboxylate (1.12 g, 3.41 mmol) in THF (75 mL) was slowly added 2 M borane-methyl sulfide complex in THF (5.2 mL, 10.4 mmol, 3.0 eq.) at 0 °C. The resulting homogenous solution was heated under reflux overnight. LCMS showed complete conversion to a single product. The reaction mixture was cooled to 0 °C, slowly quenched with water and then diluted with EtOAc. The organic layer was washed with brine, dried over MgSO_4_ and concentrated under reduced pressure. The residue was suspended in hot EtOAc (20 mL), sonicated, cooled to room temperature and filtered to afford the title compound as a light yellow solid (0.63 g). The filtrate was concentrated, taken up in EtOAc (15 mL), sonicated, heated to boiling, cooled to room temperature, filtered and washed with 50% EtOAc/heptane to give a second batch of the title compound (0.26 g). Total = 0.89 g, 89% yield. mp >225 °C. ^1^H NMR (400 MHz, DMSO-*d*_6_) δ: 11.07 (1 H, s), 10.94 (1 H, s), 8.30 (1 H, d, J = 7.8 Hz), 8.19 (1 H, d, J = 7.8 Hz), 8.06 (1 H, s), 7.75 (2 H, dd, J = 18.9, 7.8 Hz), 7.38 (2 H, m), 7.13 (2 H, q, J = 7.3 Hz), 5.43 (2 H, d, J = 5.0 Hz), 5.33 (1 H, t, J = 5.0 Hz). ^13^C NMR (100 MHz, DMSO- *d*_6_) δ: 141.6, 141.6, 135.8, 134.3, 125.9, 125.4, 123.8, 123.0, 122.9, 122.9, 121.2, 120.6, 118.1, 118.0, 117.0, 111.1, 110.6, 100.1, 58.2. LCMS m/z: [M + H]^+^  = 287.29, rt = 0.78 min.

### 6-Formylindolo[3,2-b]carbazole (FICZ, 1)

Into a solution of (5,11-dihydroindolo[3,2-b]carbazol-6-yl)methanol (0.63 g, 2.20 mmol) in 1,4-dioxane (50 mL) was added DDQ (0.55 g, 2.42 mmol, 1.1 eq.). The resulting mixture was stirred at room temperature overnight. The solid was collected by filtration, washed with dioxane, suspended in water (125 mL) and saturated sodium bicarbonate (75 mL) and stirred at room temperature for 2 h. The mixture was sonicated to break up any large blocks of solid. The solid was collected by filtration, washed with water and dried in a vacuum oven (~60 °C) to afford the first batch of FICZ as a yellow solid (0.39 g). The filtrate from the first collection was concentrated under reduced pressure, partitioned between EtOAc and 5% NaHCO_3_ and stirred overnight. The organic layer was separated and the aqueous was extracted with EtOAc two times. The combined organic was washed with 5% NaHCO_3_ and brine, dried over MgSO_4_ and concentrated. The residue was suspended in hot EtOAc, sonicated, cooled to room temperature, filtered, washed with fresh EtOAc and dried under reduced pressure to obtain a second batch of product (0.18 g). Total yield = 0.57 g, 90%. mp > 225 °C. ^1^H NMR (400 MHz, DMSO-*d*_6_) δ: 11.75 (1 H, s), 11.63 (1 H, s), 11.37 (1 H, s), 8.61 (1 H, s), 8.57 (1 H, d, J = 8.0 Hz), 8.31 (1 H, d, J = 7.5 Hz), 7.75 (1 H, d, J = 8 Hz), 7.61 (1 H, d, J = 8.3 Hz), 7.47 (2 H, dt, J = 15.6, 7.5, 7.5 Hz), 7.22 (2 H, m). ^13^C NMR (100 MHz, DMSO- *d*_6_) δ: 190.5, 142.1, 142.1, 135.8, 135.2, 126.9, 126.7, 125.1, 123.8, 122.1, 121.8, 121.5, 120.9, 119.7, 119.3, 112.8, 112.5, 111.9, 110.4. LCMS m/z: [M + H]^+^  = 285.25, rt = 0.91 min. HRMS calcd for C_19_H_12_N_2_O: 284.0950; Found: 284.0954.

### Indolo[3,2-b]carbazole-6,12-dione (13)

6-Formylindolo[3,2-b]carbazole (FICZ, 80 mg, 0.281 mmol) was suspended in MeOH (15 mL) in a 500 mL Erlenmeyer flask and covered with plastic film to prevent evaporation. The flask was irradiated in a chamber with illuminance at 11000 lux with occasional shaking. LCMS indicated very slow decomposition due to the low solubility of FICZ in MeOH. After 10 days, LCMS revealed no residual FICZ was present. The suspension was concentrated under reduced pressure. The residue was suspended in CHCl_3_, stirred and filtered. The residue that was insoluble in CHCl_3_ was collected, washed with CHCl_3_, and dried to yield the title compound as a yellow solid (40 mg, 47%). ^1^H NMR (400 MHz, DMSO-*d*_6_) δ: 12.92 (2 H, s), 8.07 (2 H, d, J = 6.8 Hz), 7.54 (2 H, d, J = 7.3 Hz), 7.38–7.28 (4 H, m). ^13^C NMR (100 MHz, DMSO- *d*_6_) δ: 176.3, 140.1, 137.9, 125.9, 124.5, 124.4, 121.8, 115.3, 114.5. LCMS m/z: [M + H]^+^  = 287.16, rt = 0.80 min.

### Photostability test

Method 1: FICZ (15.0 mg) and maleic acid (internal NMR standard, 6.0 mg) were dissolved in 5 mL of DMSO-*d*_6_. The solution was analyzed by ^1^H NMR as time point zero. 0.5 mL of the solution was placed into separate ICH 20 mL Flint clear glass bottles that were irradiated in a photostability chamber with illuminance set to 11000 lux as measured by a digital light meter. The contents of separate bottles were checked by ^1^H NMR at 1 h, 2 h, 3 h, 4 h, 5 h, 7 h, 10 h, 16 h, and 24 h. Residual FICZ was quantified by the peak height of the CHO proton (11.37 ppm, Fig. S1 peak b) compared to the height of the vinyl proton of maleic acid (6.27 ppm, Fig. S1 ref.). Decomposition of FICZ was also monitored by LCMS as additional confirmation of the NMR quantification. A parallel experiment was run under the same conditions with ICZ (12.4 mg) and maleic acid (6.4 mg) in 5.1 mL of DMSO-*d*_6_ and stability data collected at 1 h, 2 h, 4 h, 6 h, 12 h, 18 h, and 24 h.

Method 2: FICZ (8.5 mg) and maleic acid (3.5 mg) were dissolved in 4 mL of DMSO-*d*_6_. The solution was analyzed by ^1^H NMR as time point zero. 0.5 mL of the solution was placed into separate ICH 20 mL Flint clear glass bottles that were placed in direct sunlight with illuminance of 63000 lux as measured by a digital light meter (from 1:00 PM to 3:40 PM on March 17, 2015, a sunny day with minimal clouds in Durham, NC, USA). The contents of separate bottles were checked by ^1^H NMR at 1.5 min, 3 min, 10 min, 30 min, and 93 min. Residual FICZ was quantified by the peak height of the CHO proton (11.37 ppm) compared to the height of the vinyl proton of maleic acid (6.27 ppm).

Method 3: FICZ (14.4 mg) and maleic acid (internal NMR standard, 6.4 mg) were dissolved in 5 mL of DMSO-*d*_6_. The solution was analyzed by NMR as time point zero. The solution was then divided into four ICH 20 ml Flint clear glass bottles: Bottle A was exposed to air and was unprotected from the bright light; Bottle B: was flushed with nitrogen and left unprotected from the bright light; Bottle C was exposed to air but covered by aluminum-foil; and Bottle D was flushed with nitrogen and covered by aluminum foil. All bottles were placed in a photostability chamber with illuminance set to 11100 lux. The contents of each bottle were checked by ^1^H NMR at 1 d, 4 d, 8 d, 14 d and 21 d using the quantification procedure described in Method 1.

### Biological assay

CYP1A1-bla-LS180 cells (Invitrogen, catalog number K1678) were suspended in assay media (OptiMEM, 1% charcoal stripped serum, 1 mM sodium pyruvate, 0.1 mM NEAA) at a density of 20000 cells/mL. The compounds were dissolved in DMSO at a concentration of 10 mM. 0.5 mL of the compound solutions were added to a 384-well black, clear bottom, cell-coat assay plate (Griener Bio-one). A serial dilution at a ratio of 1:3 was made across the plate using assay media. A 50 μL aliquot of cell suspension was added to each well, and the compound plate was incubated overnight at 37 °C in 5% CO_2_. The plate was equilibrated at room temperature for 30 min before adding 10 μL of the LiveBLAzer™ FRET B/G substrate solution (Life Technologies) to each well. The plate was incubated for 90 min at room temperature in the dark before being read on a Perkin Elmer Envision using the bottom read fluorescence program with excitation at 400 nm and detection at 460/535 nm. Results for each test well were expressed as % inhibition using DMSO as the 0% control and 1 µM FICZ as the 100% control. In control experiments the standard AhR agonist TCDD demonstrated an EC_50_ = 0.31 nM, consistent with the vendor’s protocol.

## Supplementary information

Supplementary Information
