# Supplementary material for: Gram-scale synthesis of FICZ, a photoreactive endogenous ligand of the aryl hydrocarbon receptor
Source: Sci Rep. 2019 Jul 10;9:9982. doi: 10.1038/s41598-019-46374-7 (PMC6620467; doi:10.1038/s41598-019-46374-7)
Supplement: Supplementary file 1 — Supplementary Information [file 41598_2019_46374_MOESM1_ESM.docx]

**Supplementary Information**

**Gram-scale synthesis of FICZ, a photoreactive endogenous ligand of the**

**aryl hydrocarbon receptor**

Cunyu Zhang^1^, Katrina L. Creech^1^, William J. Zuercher^2^, and Timothy M. Willson^2^*

^1^ Platform Technology Sciences, GlaxoSmithKline, Collegeville, PA, USA

^2^ Structural Genomics Consortium, UNC Eshelman School of Pharmacy, University of North Carolina at Chapel Hill, Chapel Hill, NC, USA

* Corresponding author

E-mail tim.willson@unc.edu (TW)

**
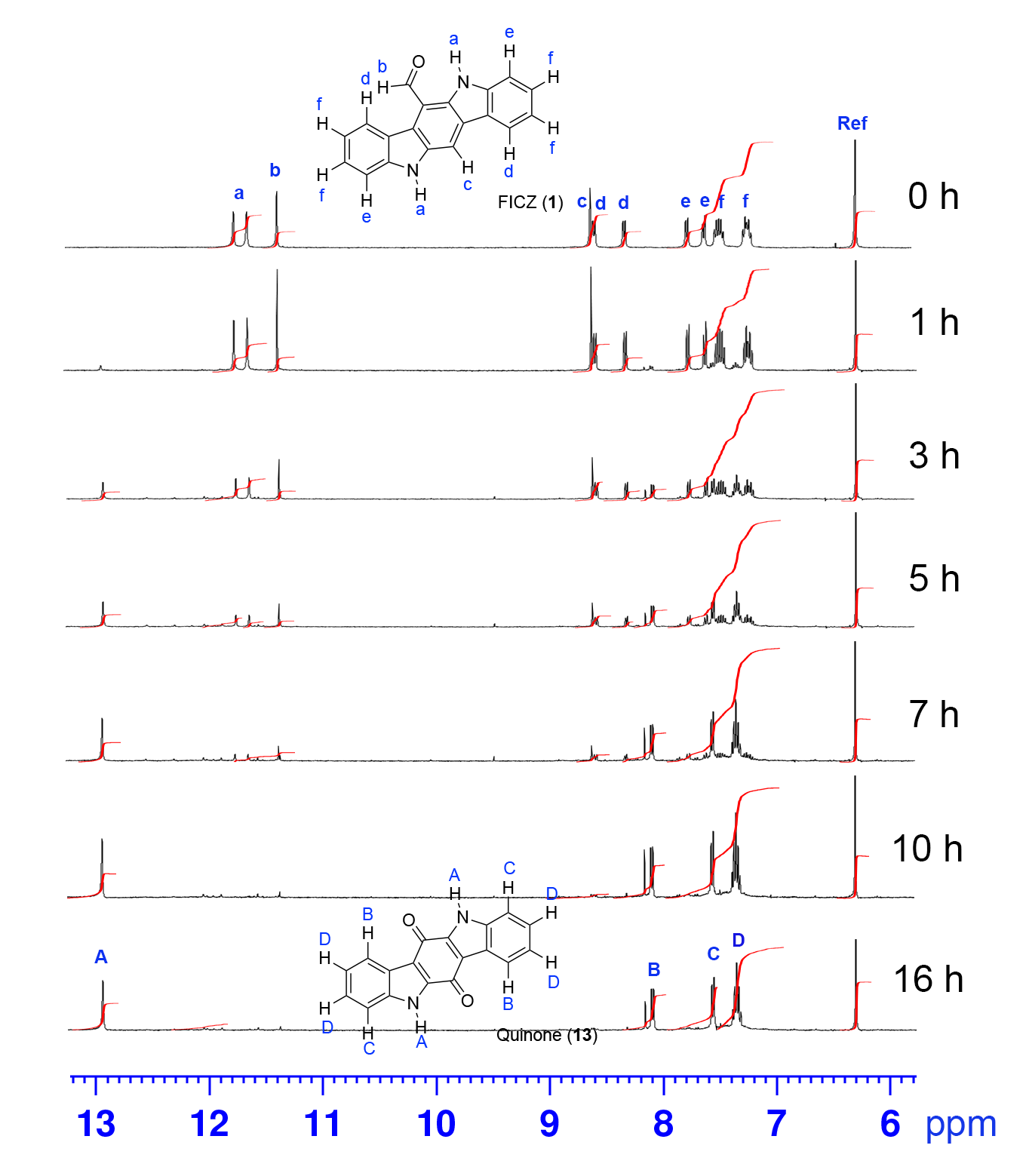
**

**Figure S1.** Time course of the photo-reaction of FICZ (**1**). The reaction was monitored by ^1^H NMR as described in Method 1 (*Materials and methods*). The ^1^H NMR spectrum at 0 h is labeled with the proton assignments for FICZ (**1**). The ^1^H NMR spectrum at 16 h is labeled with the proton assignments for quinone (**13**). No other carbazoles were observed as byproducts or intermediates. There was a minor unassigned singlet at 8.17 ppm. Ref indicates the maleic acid internal standard.

**Figure S2.** Proposed mechanism for the formation of quinone (**13**) from either FICZ (**1**) or ICZ (**12**). The mechanism is based on the radical chain pathway for photooxidation of benzaldehyde^1^. The aldehyde in FICZ (**1**) facilitates the critical initiation step of the radical chain. Peroxide radical is shown as the initiator, but could be any trace oxygen radical. 6-Carbonyl radical (A) captures oxygen to produce a carbazole 6-carboperoxy radical (B) that further propagates the chain by reaction with FICZ (**1**) and production of the carboxy radical (C), which in turn generates the key carbazole radical (D) through loss of CO_2_. In the absence of a 6-carbonyl substituent, initiation and propagation of the radical chain would be much slower from ICZ (**12**) but could still generate the same intermediate radicals (D or E). Subsequent conversion of the peroxy-radical intermediate (E) to quinone (**13**) could occur by multiple mechanisms^2^.

**Figure S3. Photobiology of FICZ.** Potential role of the quinone (**13**) photoproduct in the ROS-induced action of AhR. Hypothesized steps involving redox cycling to remove reactive oxygen species are shown with dashed arrows.

**Analytical spectra**

**(1-(Phenylsulfonyl)-1H-indol-2-yl)(1-(phenylsulfonyl)-1H-indol-3-yl)methanol (5)**

^1^H NMR

**Ethyl 2-(2-((1H-indol-3-yl)methyl)-1H-indol-3-yl)-2-oxoacetate (7)**

^1^H NMR

**5,11-Dihydroindolo[3,2-b]carbazole-6-carboxylic acid (9)**

^1^H NMR
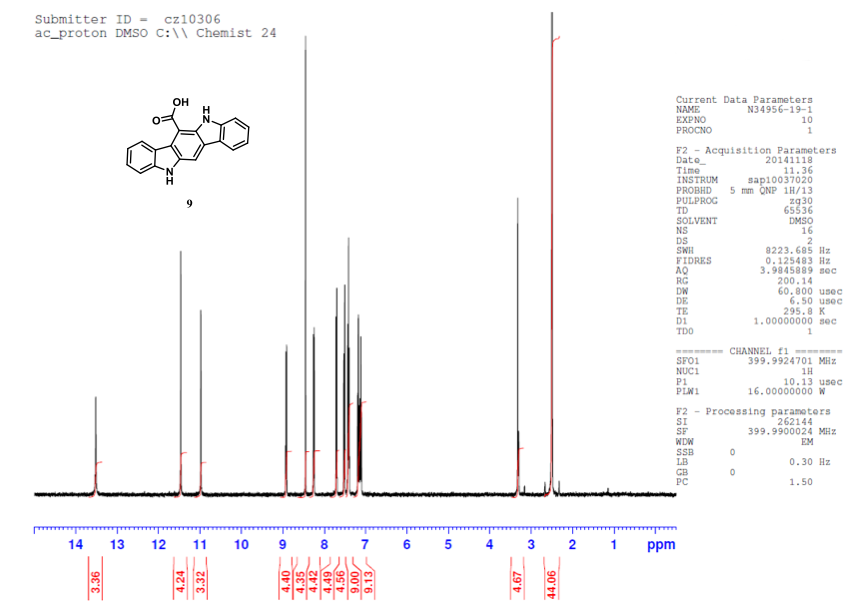


**Ethyl 5,11-dihydroindolo[3,2-b]carbazole-6-carboxylate (10)**

^1^H NMR
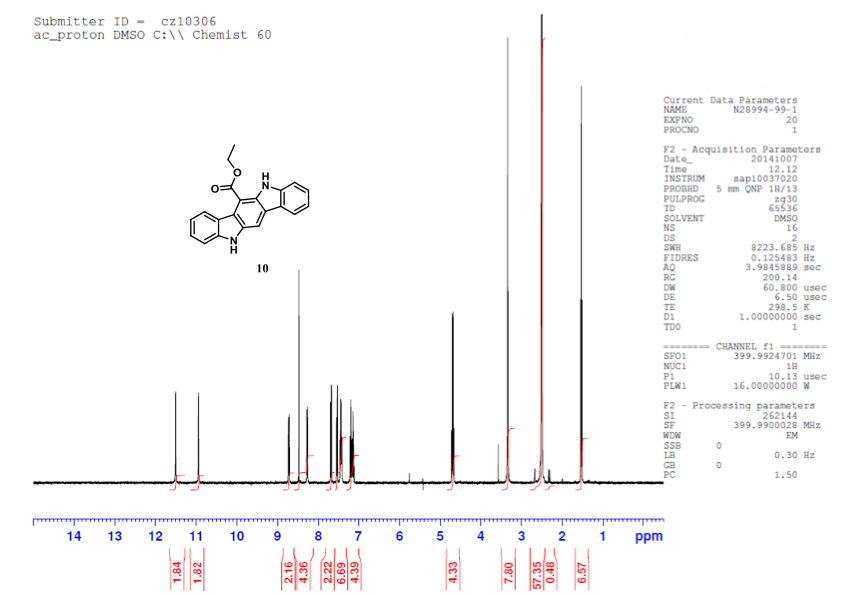


^13^C NMR
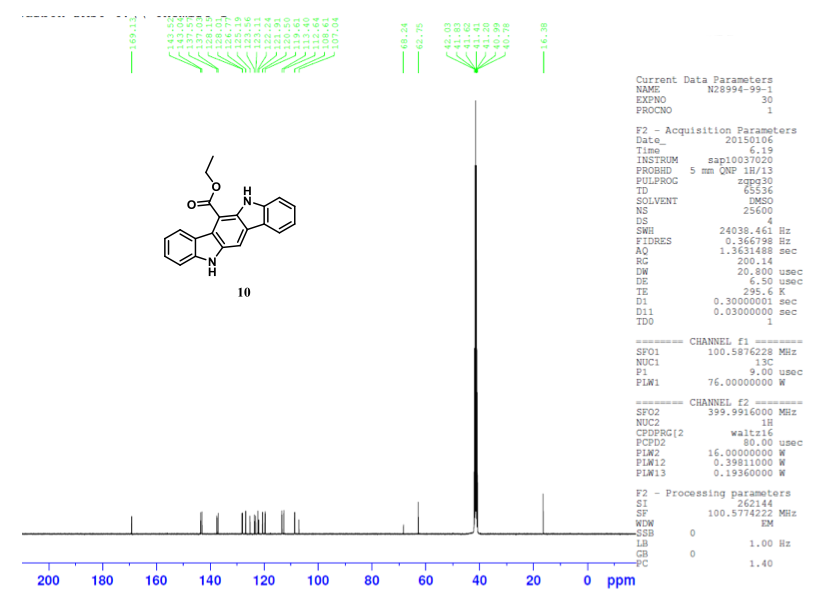


**(5,11-Dihydroindolo[3,2-b]carbazol-6-yl)methanol** **(11)**

^1^H NMR
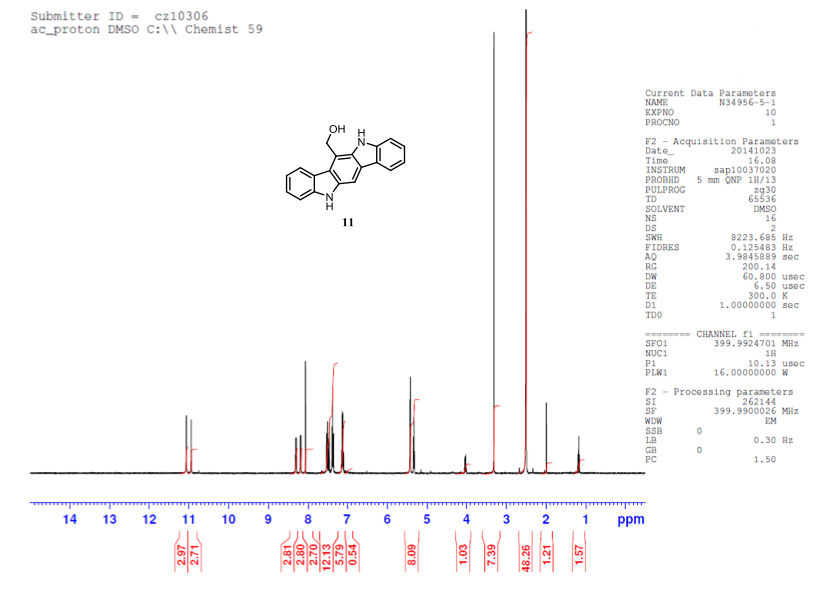


^13^C NMR
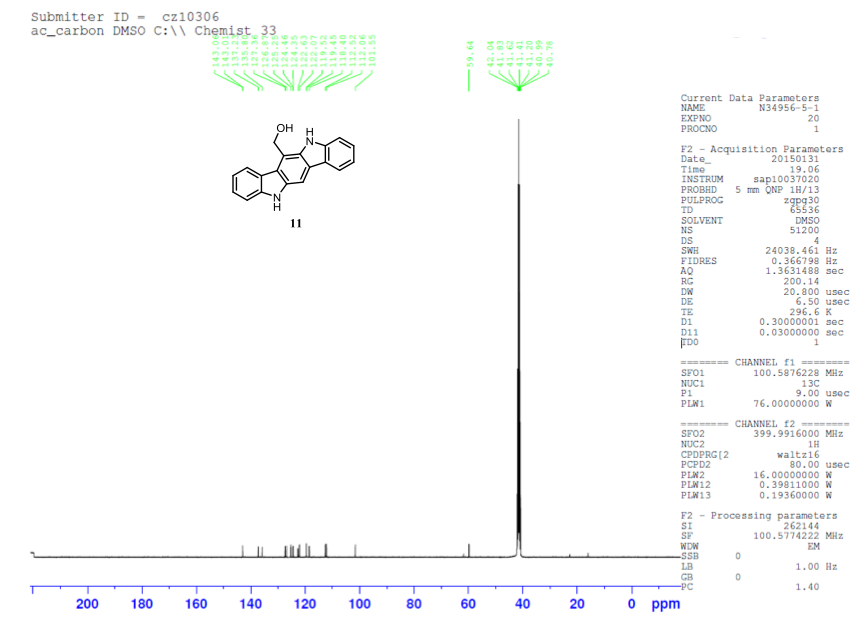


**Indolo[3,2-b]carbazole-6,12-dione (13)**

^1^H NMR

^13^C NMR
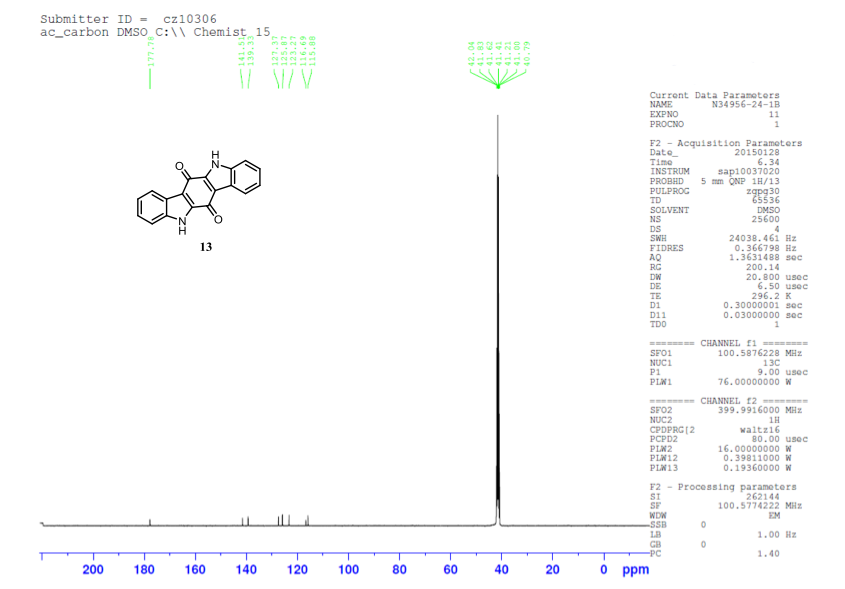


**6-Formylindolo[3,2-b]carbazole** (**FICZ, 1**)

^1^H NMR

^13^C NMR
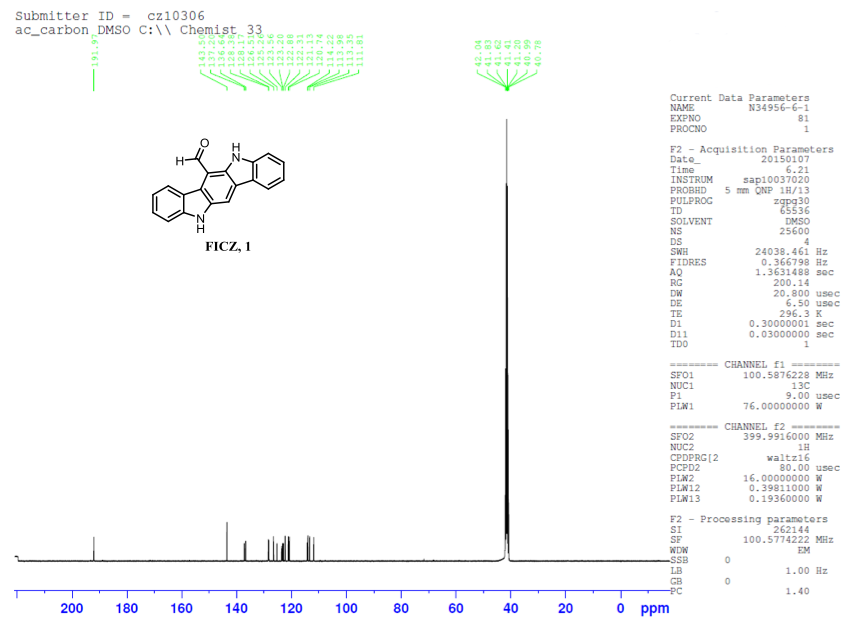


HRMS

**References**

1 Sankar, M. *et al.* The benzaldehyde oxidation paradox explained by the interception of peroxy radical by benzyl alcohol. *Nat Commun* **5**, 3332, doi:10.1038/ncomms4332 (2014).

2 Bolton, J. L. & Dunlap, T. Formation and Biological Targets of Quinones: Cytotoxic versus Cytoprotective Effects. *Chem Res Toxicol* **30**, 13-37, doi:10.1021/acs.chemrestox.6b00256 (2017).
